# The search for stable prognostic models in multiple imputed data sets

**DOI:** 10.1186/1471-2288-10-81

**Published:** 2010-09-17

**Authors:** David Vergouw, Martijn W Heymans, George M Peat, Ton Kuijpers, Peter R Croft, Henrica CW de Vet, Henriëtte E van der Horst, Daniëlle AWM van der Windt

**Affiliations:** 1 Institute for Research in Extramural Medicine, VU University Medical Center Amsterdam, The Netherlands; 2 VU University, Institute for Health Sciences, Department of Methodology and Applied Biostatistics, Amsterdam, The Netherlands; 3 Primary Care Musculoskeletal Research Centre, Keele University, Keele Staffordshire, UK; 4 Dutch Institute for Healthcare Improvement CBO, Utrecht, The Netherlands

## Abstract

**Background:**

In prognostic studies model instability and missing data can be troubling factors. Proposed methods for handling these situations are bootstrapping (B) and Multiple imputation (MI). The authors examined the influence of these methods on model composition.

**Methods:**

Models were constructed using a cohort of 587 patients consulting between January 2001 and January 2003 with a shoulder problem in general practice in the Netherlands (the Dutch Shoulder Study). Outcome measures were persistent shoulder disability and persistent shoulder pain. Potential predictors included socio-demographic variables, characteristics of the pain problem, physical activity and psychosocial factors. Model composition and performance (calibration and discrimination) were assessed for models using a complete case analysis, MI, bootstrapping or both MI and bootstrapping.

**Results:**

Results showed that model composition varied between models as a result of how missing data was handled and that bootstrapping provided additional information on the stability of the selected prognostic model.

**Conclusion:**

In prognostic modeling missing data needs to be handled by MI and bootstrap model selection is advised in order to provide information on model stability.

## Background

In healthcare predicting how long it takes for an episode of musculoskeletal pain to resolve can be difficult. Outcome varies between patients and over time. Although clinicians can be relatively good "prognosticians" [1,2] clinical judgment and intuition can be incorrect and difficult to quantify or to be made explicit. To understand the ingredients that contribute to correct prognosis and to improve upon clinical judgment, clinical prediction rules can be useful. These provide a quantitative estimate of the absolute risk of particular outcomes of interest for individual patients, which may subsequently be used to support decisions regarding treatment. Until now, several clinical prediction rules have been developed in the field of musculoskeletal pain, for example to estimate the outcome of low back [2-4], knee [5] or shoulder pain [6].

In the development of clinical prediction models, researchers frequently use a regression analysis with a backward or forward selection strategy. However, this methodology may result in overoptimistically estimated regression coefficients, omission of important predictors and random selection of less important predictors. As a result derived models may be unstable [7]. Incorporating a bootstrap resampling procedure in model development has been suggested to provide information on model stability [8-11]. Since bootstrapping mimics the sampling variation in the population from which the sample was drawn it is expected to produce a model which better represents the underlying population [9-11].

Another problem occurring in prognostic studies is missing data. Multiple imputation (MI), which uses all observed information, was shown to be superior to other imputation techniques like single regression imputation [12,13]. Though, MI is not yet frequently used in predictive modelling and model stability is hardly ever accounted for in MI approaches. It has been shown that for low back pain extending MI with a bootstrapping procedure provides an accurate model selection and information on model stability [14]. However generalizability of this method was never tested in other patient datasets.

Therefore, the objective of our research was to examine the influence of bootstrapping and multiple imputation on model composition and stability in a shoulder pain data set with missing values.

## Methods

### Study population

We used data from the Dutch Shoulder Study (DSS) [15]. This cohort consists of 587 patients who consulted their general practitioner (GP) with a new episode of shoulder disorders. Inclusion criteria were: no GP consultation or treatment received for the afflicted shoulder in the preceding three months. Exclusion criteria were: dementia, severe psychiatric of physical conditions (i.e. fractures or dislocation in the shoulder region, rheumatic diseases, neoplasms, neurological of vascular disorders). The ethics review board of the VU University medical centre approved the study protocol.

### Outcome measures

We focused on two outcome measures; persistent shoulder disability (16-item SDQ; 0-100) [16] and persistent shoulder pain intensity (Numeric Rating Scale; 0-10) [17]. To define 'persistence' baseline scores were subtracted from follow-up scores. An optimal cut-off point was defined by studying the relationship between the change scores and a secondary outcome measure 'patient perceived recovery' [6]. Patients were denoted as recovered when they characterized their complaints as 'fully recovered' or 'very much improved'. By constructing Receiver Operating Characteristic (ROC) curves with patient perceived recovery as the external criterion, the optimal cutoff point (i.e. that point that yields the lowest overall misclassification) was determined [18]. According to this analysis a 50% decrease in disability and pain intensity compared to baseline was considered a minimal important change, and was used as a cut-off value to dichotomize both outcome measures. Patients who improved less then 50% were denoted as having persistent pain or disability. Outcomes were measured three months after enrolment by postal questionnaire.

### Prognostic factors

Based on a systematic review of the literature [19] a set of candidate predictors was selected, including demographic variables, characteristics of the shoulder pain problem, physical and psychological factors (see Table 1). The following questionnaires were used to gather information on psychological factors: the Pain Coping and Cognition List (PCCL [20]: pain coping, catastrophizing, internal and external locus of control), the 4 Dimensional Symptom Questionnaire (4DSQ [21]: anxiety, depression, somatisation, distress), the Fear-Avoidance Beliefs Questionnaire (FABQ [22]: fear-avoidance) and the Tampa Scale for Kinesiophobia (TSK [23,24]: kinesiophobia). Within 10 days after consulting the GP, participants completed a baseline questionnaire to assess potential predictors.

**Table 1:** Patient characteristics at baseline

variable	n	(%)
**Demographic**		
Age (years); mean (SD)	51	(14)
gender (male)	292	(50)
education		
low*	210	(36)
middle	234	(40)
high	135	(23)
**Disease characteristics**		
shoulder complaints in the past year	321	(55)
neck complaints in the past year	252	(43)
duration of complaints		
0-6 weeks	205	(35)
7-12 weeks	139	(24)
> 3 months	242	(41)
gradual onset (vs. acute)	363	(62)
shoulder pain (0-10); mean (SD)	4.8	(2)
shoulder disability (0-100); mean (SD)	59.9	(24)
both shoulders afflicted	74	(13)
co-morbidity	469	(80)
upper extremity joint pain	245	(42)
neck pain	197	(34)
upper extremity joint pain	174	(30)
low back pain	139	(24)
high back pain	53	(9)
**Psychological factors**		
psychological complaints	55	(9)
pain coping (0-6); mean (SD)	2.98	(0.98)
catastrophizing (0-6); mean (SD)	2.2	(0.8)
internal locus of control (0-6); mean (SD)	3.3	(0.9)
external locus of control(0-6); mean (SD)	3.2	(0.88)
anxiety (0-6); mean (SD)	0.3	(1.2)
depression (0-6); mean (SD)	0.2	(1.3)
somatisation (0-6); mean (SD)	3.3	(4.1)
distress (0-6); mean (SD)	2.3	(4.5)
fear-avoidance (0-6); mean (SD)	14.1	(5.6)
kinesophobia (0-6); mean (SD)	3.2	(3.5)
**Physical factors**		
physical load at work (0-5); mean (SD)	1.2	(1.5)
physical activity		
less active than others	110	(39)
equally active	245	(42)
more active	226	(19)
inability to perform daily activities last year		
1-30 days	184	(31)
1-12 months	61	(10)
sporting activities	230	(39)
cause of shoulder problem: sporting injury	29	(5)

SD = standard deviation

### Analysis

For all continuous predictors the linearity assumption was checked. When the relationships between variables and outcome did not resemble linearity, variables were categorized (3 categories) or dichotomized. Although this causes loss of information [25], these procedures were retained since they are part of the frequently used standard statistical methodology in predictive modeling. Variables were checked for (multi)collinearity using Pearson's r, given that correlated variables can disturb variable selection in multivariable regression [26]. In case of correlation (r≥0.5) the variables which could most easily be obtained in clinical practice by the physician were retained.

To reduce the initial number of variables, an univariable analysis (α > 0.157) was performed in both the imputed and unimputed data sets, thus all analyses were preceded by this pre-selection. The subsequent analyses were all based on a multivariable analysis with a backward selection strategy and a stopping rule of α = 0.157. This significance level is available in many statistical software packages and results have been shown to be comparable with the more complex Akaike Information Criterion (AIC) [27]. The number of events per variable (EPV) was calculated for each method to check whether the analysis was sufficiently powered (EPV > 10) [28]. The checklist proposed by Harrell [29] for multivariable modeling was followed where possible. To study the effect of missing data and model stability on model composition, the following four methods were compared:

#### 1) Complete Case Analysis (CCA)

To handle missing data, subjects with missing values on any of the variables were omitted and only those subjects with information on all variables in the model were included for analysis.

#### 2) Multiple imputation (MI-5)

Missing values were imputed using a Multivariate Imputation by Chained Equations (MICE) procedure with the "predictive mean matching" as imputation method [30]. All available data including outcome measure were used in the imputation method [13]. We generated five imputed data sets (MI-5). Multivariable regression was applied to each of the 5 imputed data sets. From these 5 models, predictors which appeared in at least 2 models (a Inclusion Fraction of ≥40%) qualified for the final model. Whether these predictors significantly contributed to the final model was tested using a likelihood ratio test [31] with a critical *P*-value of *P *= 0.157. Predictors were dropped from the final model in case of a nonsignificant (*P *> 0.157) likelihood ratio.

#### 3) Bootstrapping (B)

A two-step bootstrap model selection procedure [9,11] was applied to provide information on model stability. First 500 samples with replacement were taken from the complete case data set. In each sample a multivariable model was built. To be consistent with the MI-5 method, predictors which appeared in ≥40% of these models qualified for the second step. In this second step 500 new complete case samples were taken and in each of which a multivariable model was built using the predictors from the first step. These 500 models provided information on model stability (i.e. which combination of predictors is most frequently selected in the model).

#### 4) Multiple imputation + bootstrapping (MI-5+B)

Missing data was imputed using the MICE procedure and five imputed data sets were created. In each of the five imputed data sets the two step bootstrap model selection procedure as described above was applied. Information on model stability was provided by studying which combination of predictors occurred most frequently in 2500 data sets.

### Internal validation

The apparent performance of a predictive model is typically better in the data set in which the model has been developed compared to its performance in another similar data set [32]. This phenomenon is called overoptimism. Using a n = 200 samples bootstrap procedure for internal validation [33] the performance of each developed model was tested in similar populations as in the derivation sample. This method was used to estimate the overoptimism of the derived models, and to adjust the measures of performance.

### Model evaluation

Derived models were evaluated by comparing the model's composition (combination of predictors). Next several measures of predictive performance were considered. Discrimination refers to how well a model distinguishes between patients with and without persistent symptoms and is quantified by the c-index that, for binary outcomes, is identical to the area under the ROC curve (AUC) [34]. The c-index varies between 0.5 and 1, with 0.5 indicating no discrimination above chance and 1 indicating perfect discrimination. The agreement between predicted probabilities and observed probabilities is called calibration and was measured by computing the slope of the calibration plot (predicted probabilities against observed frequencies). Well-calibrated models have a slope of 1. As a measure of the explained variance Nagelkerke's R^2 ^was computed.

### Software

All analyses were performed using the R-statistics software (version 2.4.0). The R Design package was used for the CCA, MICE was used for the MI and additional routines were developed for applying the bootstrap.

## Results

The baseline patient characteristics are listed in Table 1. After three months 517 patients (88%) returned the follow-up questionnaire. Subjects lost to follow-up were younger (mean difference of 7 years) and showed more often an acute onset (47% versus 37%). Due to non-response the percentage of missing data was largest for the outcome measures (shoulder disability 12.3% and shoulder pain intensity 12.9%). Other (baseline) variables had missing values within the range of 0 to 9.2%. The combination of missing values in CCA resulted in the exclusion of 24.7% (disability model) and 28.8% (pain intensity model) of participants.

In the CCA 12 variables showed a univariable association with persistent disability and 16 with persistent pain, resulting in an EPV of 11.9 for pain intensity and 17 for shoulder disability. In the five imputation data sets the EPV varied between 19.1 and 19.6 for disability and between 13.5 and 13.8 for pain intensity. This means that the analyses were sufficiently powered (with a sufficient number of cases in the models) to reliably estimate the associations between predictors and outcome.

### Model composition

For all presented models, the directions of the associations (i.e. regression coefficients) between the selected predictors and outcome were the same for both disability and pain (data not presented). Tables 2 and 3 show that for both measures of outcome, model composition was influenced by missing data (CCA vs. MI-5). When models were derived from imputed data, model composition diverged from the CCA model. For both measures of outcome predictors with lower predictive abilities in the CCA (i.e. rank order according to regression coefficient estimates) were not included in the MI-5 (e.g. concomitant lower extremity pain for shoulder disability and for pain intensity; sporting activities and higher physical workload). Predictors that were no part of the CCA model entered the MI-5 model for persistent shoulder disability (e.g. duration of complaints, somatisation, external locus of control and age) were included in the MI-5 model.

**Table 2:** Complete case and multiple imputed model compositions for the outcome measure persistent shoulder disability

	missing values	CCA	MI-5
		rank	rank
persistent shoulder disability*	72 (12.3%)		
inability to perform daily activities	8 (1.4%)	1	4
shoulder complaints in the past year	27 (4.6%)	2	
both shoulders afflicted	0 (0%)	3	3
concomitant lower back pain	0 (0%)	4	1
concomitant lower extremity pain	0 (0%)	5	
more disability at baseline	2 (0.3%)	6	7
longer duration of complaints	1 (0.2%)		2
higher scores for somatisation	3 (0.5%)		5
higher scores for external locus of control	33 (5.6%)		6
older age	0 (0%)		8

CCA      - complete case analysis

MI -5     - multiple imputation using 5 imputation files

rank      - the order of appearance of predictors in the derived model arranged by their predictive ability (regression coefficient)

*           - outcome measure

**Table 3:** Complete case and multiple imputed model compositions for the outcome measure persistent shoulder pain intensity

	missing values	CCA	MI-5
		rank	rank
persistent shoulder pain intensity*	76 (12.9%)		
sporting injury	0 (0%)	1	1
concomitant lower back pain	0 (0%)	2	3
longer duration of complaints	1 (0.2%)	3	2
both shoulders afflicted	0 (0%)	4	4
inability to perform daily activities	8 (1.4%)	5	5
concomitant upper extremity pain	0 (0%)	6	6
sporting activities	0 (0%)	7	
higher physical workload	0 (0%)	8	

CCA      - complete case analysis

MI -5     - multiple imputation using 5 imputation files

rank      - the order of appearance of predictors in the derived model arranged by their predictive ability (regression coefficient)

%          - inclusion frequency; the proportion of times that a variable with a univariable association with the outcome is retained in the automated backward selected models. When a variable was selected in each of the replications, the inclusion frequency was 100%

*           - outcome measure

Tables 4, 5, 6 and 7 show the results of assessing model stability by the bootstrap model selection procedure. CCA and MI-5 models were not identified as the most frequently occurring combination of predictors for both outcome measures (Tables 4, 5, 6). Only the persistent shoulder disability MI-5 method was identical to its bootstrapped enhanced version (Table 7). Model selection frequencies for the most frequently selected models were uniformly low (ranging from 24.0% to 3.6%). Indicating on a large variability in model composition within the bootstrap replicate data sets. When fewer potential predictors are retained after the first step of the bootstrap model selection procedure, this variability seemed to decrease and model selection frequency increased.

**Table 4:** Complete case bootstrap model selection for the outcome measure persistent disability

	most frequently selected models					rank	
Predictors*	1	2	2	4	5	B	CCA
inability to perform daily activities	X	X	X	X	X	1	1
both shoulders afflicted	X	-	-	X	X	2	3
shoulder complaints in the past year	X	X	X	X	-	3	2
concomitant lower extremity pain	X	X	X	X	X	4	5
more disability at baseline	X	X	X	X	X	5	6
concomitant lower back pain	-	-	X	X	X	-	4
older age	-	-	-	-	-	-	-
longer duration of complaints	-	-	-	-	-	-	-
acute onset	-	-	-	-	-	-	-
							
Count	33	23	23	22	16		
%	6.6	4.6	4.6	4.4	3.2		

*            - only those predictors that appeared in ≥40% of the first bootstrap model selection step are presented

rank       - the order of appearance of predictors in the derived models arranged by their predictive ability (regression coefficient estimates)

B           - the complete case date based bootstrap selected model (i.e. the most frequently occurring combination of predictors in 500 replicate data sets of the second bootstrap model selection step)

CCA       - the complete case data based model derived without additional bootstrap was the fourth most occurring combination of predictors in the bootstrap model selection procedure

Count     - the number of times the model was selected in the 500 replicate data sets of the second bootstrap model selection step

**Table 5:** Complete case bootstrap model selection results for the outcome measure persistent pain intensity

	most frequently selected models					rank	
Predictors	1	2	3	4	5	B	CCA
longer duration of complaints	X	X	X	X	X	1	3
concomitant lower back pain	X	X	X	X	X	2	2
both shoulders afflicted	X	X	-	X	X	3	4
concomitant upper extremity pain	X	X	X	-	-	4	6
shoulder complaints in the past year	X	-	X	X	-	5	-
sporting injury*						-	1
inability to perform daily activities*						-	5
sporting activities*						-	7
higher physical workload*						-	8
							
Count	120	96	58	47	37		
%	24.0	19.2	11.6	9.4	7.4		

*             - predictors that appeared in ≥ 40% in the first step of the of the bootstrap model selection are not used in the second step in model selection

rank        - the order of appearance of predictors in the derived models arranged by their predictive ability (regression coefficient estimates)

B            - the complete case date based bootstrap selected model (i.e. the most frequently occurring combination of predictors in 500 replicate data sets of the second bootstrap model selection step)

CCA        - the complete case data based model derived without additional bootstrap did not occur in the bootstrap model selection since some of the included predictors occurred ≥ 40% in the first selection step

Count     - the number of times the model was selected in the 500 replicate data sets of the second bootstrap model selection step

**Table 6:** Imputed bootstrap model selection results for the outcome measure persistent pain intensity

	most frequently selected models					rank	
Predictors*	1	2	3	4	5	MI-5+B	MI-5
sporting injury	X	X	X	X	X	1	1
longer duration of complaints	X	X	X	X	X	2	2
concomitant lower back pain	X	X	X	X	X	3	3
both shoulders afflicted	X	X	X	X	X	4	4
inability to perform daily activities	X	X	X	X	X	5	5
higher level of education	X	X	X	-	X	6	-
shoulder complaints in the past year	X	X	-	-	X	7	-
concomitant upper extremity pain	X	-	X	X	X	8	6
higher physical workload	X	X	X	-	X	9	-
							
Count	163	158	113	111	105		
%	6.5	6.3	4.5	4.4	4.2		

*             - only those predictors that appeared in ≥40% of the first bootstrap model selection step are presented

rank        - the order of appearance of predictors in the derived models arranged by their predictive ability (regression coefficient estimates)

MI-5+B    - the multiple imputation based bootstrap selected model (i.e. the most frequently occurring combination of predictors in 2500 replicate data sets of the second bootstrap model selection step)

MI-5        - the multiple imputation based model using 5 imputed data sets was the fourth most occurring combination of predictors in the bootstrap model selection procedure.

Count      - the number of times the model was selected in the 2500 replicate data sets of the second bootstrap model selection step

**Table 7:** Imputed bootstrap model selection results for the outcome measure persistent disability

	most frequently selected models					rank	
Predictors*	1	2	3	4	5	MI-5+B	MI-5
concomitant lower back pain	X	X	X	X	X	1	1
longer duration of complaints	X	X	X	X	-	2	2
both shoulders afflicted	X	X	X	X	X	3	3
inability to perform daily activities	X	X	X	-	X	4	4
higher scores for somatisation	X	X	X	X	X	5	5
higher scores for external locus of control	X	X	X	X	X	6	6
more disability at baseline	X	X	X	X	X	7	7
older age	X	X	-	X	X	8	8
shoulder complaints in the past year	-	X	X	-	X	-	-
concomitant lower extremity pain	-	-	-	-	-	-	-
							
Count	91	77	56	54	52		
%	3.6	3.1	2.2	2.2	2.1		

*             - only those predictors that appeared in ≥40% of the first bootstrap model selection step are presented

rank        - the order of appearance of predictors in the derived models arranged by their predictive ability (regression coefficient estimates)

MI-5+B    - the multiple imputation based bootstrap selected model (i.e. the most frequently occurring combination of predictors in 2500 replicate data sets of the second bootstrap model selection step)

MI-5        - the multiple imputation based model using 5 imputed data sets was also the most frequently occurring combination of predictors in the 2500 bootstrap replicate 	data sets

Count      - the number of times the model was selected in the 2500 replicate data sets of the second bootstrap model selection step.

### Model performance

Table 8 presents the performance of the models derived with the four methods for both outcome measures. The slopes of the calibration plots ranged from 0.973 to 1.077, which indicates good calibration. Explained variance ranged from 8.8% to 12.0% for disability and from 13.5% to 18.8% for pain. The apparent c-indices varied between 0.645 and 0.667 for disability and between 0.684 and 0.717 for pain intensity. CCA models were more optimistic compared to the other models. Following adjustment for overoptimism the corrected c-indices were within the range of 0.639 - 0.646 for persistent shoulder disability and within the range of 0.667 - 0.688 for persistent shoulder pain.

**Table 8:** Model performance parameters.

Persistent disability					Persistent shoulder pain intensity			
	CCA	MI-5	B	MI-5 + B	CCA	MI-5	B	MI-5 + B
calibration slope	0.978	0.978	1.077	0.978	0.985	0.973	0.998	0.986
R^2^_N_	0.119	0.120	0.088	0.120	0.188	0.162	0.135	0.174
Ac 95% CI	0.666 0.616,0.715	0.667 0.624,0.710	0.645 0.596,0.694	0.667 0.624,0.710	0.717 0.668,0.766	0.702 0.660,0.745	0.684 0.637,0.732	0.710 0.668,0.752
Opt	0.027	0.022	0.023	0.022	0.030	0.014	0.018	0.022
Oc	0.639	0.646	0.622	0.646	0.686	0.688	0.667	0.688

Ac               - apparent c-index

B                - bootstrapping based on a complete case data set

CCA            - complete case analysis

MI-5+B        - multiple imputation combined with bootstrapping

MI-5            - multiple imputation using 5 imputation files

Oc              - optimism corrected c-index

Opt             - estimation of the overoptimism

R^2^_N_            - explained variance (Nagelkerke's R-squared)

95% CI        - 95% confidence interval

## Discussion

Prognostic research aims at identifying the ingredients that contribute to a correct prognosis for a specific subgroup of patients. Though, finding a stable set of predictors that can consistently be used in a broad patient population proves to be difficult. Several methodological issues (missing data and model stability) which are not accounted for by the standard statistical methodology are expected to complicate this matter. We showed that accounting for missing data by MI and providing information on model stability by bootstrapping are instructive methods when deriving a prognostic model.

In the standard statistical methodology the use of a backward or forward selection strategy has been criticized. It may result in overoptimistically estimated regression coefficients, omission of important predictors and random selection of less important predictors. Derived models may therefore be unstable. Research has focussed on how to derive stable models. One frequently used method is the bootstrapping approach suggested by Austin and Tu [35]. It considers the strength of evidence that identified variables are truly important predictors in re-sampled data. Although this approach is often claimed to reduce model instability [8,10,14,35,36], separating strong from weak predictors was shown to perform comparative to automated backward elimination in identifying the true regression model [37]. Furthermore, this approach has limited abilities when there is a high number of potential prognostic factors. For these situations a modified bootstrapping procedure was suggested [11]. Our study showed that the application of this two-step bootstrap model selection procedure provides valuable information on model stability.

As frequently described, model size and model composition are also affected by missing data. Especially in standard statistical methodology where subjects with missing values on any of the recorded variables are omitted from analysis. When missing data does not depend on observed or unobserved measurements (Missing Completely At Random, MCAR), this leads to loss of costly gathered information, decreased statistical power, altered associations between predictors and therefore differences in model composition [12,13,38-41]. In this context our study findings formed no exception. Model composition varied as a result of whether cases with missing data were omitted from analyses (CCA) or whether the values of the missings were estimated using MI. Since missing values appeared to be related to other observed information, the MCAR condition did not hold and CCA was expected to be biased. Most of the missing data was observed in the outcome because participants did not consent to follow-up. As subjects lost to follow-up showed more often an acute onset (47% versus 37%), were younger (mean difference of 7 years) and the variable age is included in the MI model for the outcome measure persistent shoulder disability, it is plausible to assume that these missings are MAR. For that reason, accounting for missing data by MI using 5 imputed data sets was in our multivariate data setting the most optimal choice to reduce the uncertainty in model derivation caused by missing values. The use of even more data sets in the imputation routine is possible (up to 20), however 5 was shown to be an sufficient number in order to get stable results [30]. Yet the addition of a bootstrap model selection procedure showed that the MI-5 model might still be unstable. A possible source for this instability might be the suboptimal variable selection procedure applied in the MI-5 procedure. However, how to optimally perform variable selection in multiple imputed data is still a subject of discussion [42]. As illustrated by our study, the bootstrap model selection procedure may provide valuable additional information on model stability when deriving a prognostic model in multiple imputed data.

To study the effects of accounting for missing data and incorporating model stability we used a large clinical data set in which we empirically evaluated different methods of deriving a prognostic model. By this, the uncertainties researchers commonly face when knowledge of the true predictors of outcome is lacking, were illustrated. Furthermore, the practical utility of the additional information provided by the bootstrap model selection procedure in prognostic modeling is demonstrated. Though results need to be interpreted with caution, as our approach limits us from identifying a superior methodology. Although performance parameters for each derived model are presented, these play no role in the decision on the superiority of a certain method. They only show that the performance of all derived models was comparable to that from existing clinical prediction rules on shoulder pain [6,15]. For deciding on the superiority of a certain method, a simulation study in which true predictors and noise variables are assigned would be needed. Such data is not presented by this study.

## Conclusions

Our study showed that in this particular dataset of shoulder pain patients, model composition varied as a result of how missing data was handled. Furthermore, the bootstrap model selection routine gave additional information on model stability.

## Competing interests

The authors declare that they have no competing interests.

## Authors' contributions

Authors DvdW and TK were responsible for the conception, design and data collection of the study. Author MH designed the study's analytic strategy and helped to prepare the Material & Methods and the Discussion sections. Authors PC, GP, HvdH, HdV, MH, and DvdW critically appraised and revised draft versions of the manuscript. Author DV prepared the data for analyses, designed the analytic strategy, conducted all analyses and wrote the paper. All authors read and approved the manuscript.

## Pre-publication history

The pre-publication history for this paper can be accessed here:

http://www.biomedcentral.com/1471-2288/10/81/prepub

## References

[B1] Schiøttz-Christensen B Nielsen GL Hansen VK Schødt T Sørensen HT Olesen F Long-term prognosis of acute low back pain in patients seen in general practice: a 1-year prospective follow-up study Fam Pract 1999 16 223 32 10.1093/fampra/16.3.223 10439974

[B2] Jellema P van der Windt DA van der Horst HE Stalman WA Bouter LM Prediction of an unfavourable course of low back pain in general practice: comparison of four instruments Br J Gen Pract 2007 57 15 22 17244419 PMC2032695

[B3] Heymans MW Anema JR van Buuren S Knol DL van Mechelen W de Vet HC Return to work in a cohort of low back pain patients: development and validation of a clinical prediction rule J Occup Rehabil 2009 19 155 65 10.1007/s10926-009-9166-3 19224347

[B4] Dionne CE Bourbonnais R Frémont P Rossignol M Stock SR Larocque I A clinical return-to-work rule for patients with back pain CMAJ 2005 172 1559 67 15939915 10.1503/cmaj.1041159 PMC558170

[B5] Thomas E Dunn KM Mallen C Peat G A prognostic approach to defining chronic pain: Application to knee pain in older adults Pain 2008 139 389 97 10.1016/j.pain.2008.05.010 18583051

[B6] Kuijpers T van der Windt DA Boeke AJ Twisk JWR Vergouwe Y Bouter LM van der Heijden GJMG Clinical prediction rules for the prognosis of shoulder pain in general practice Pain 2006 120 276 85 10.1016/j.pain.2005.11.004 16426760

[B7] Austin PC Tu JV Automated variable selection methods for logistic regression produced unstable models for predicting acute myocardial infarction mortality J Clin Epidemiol 2004 57 1138 46 10.1016/j.jclinepi.2004.04.003 15567629

[B8] Sauerbrei W The use of resampling methods to simplify regression models in medical statistics Applied Statistics 1999 48 313 329

[B9] Sauerbrei W Schumacher M A bootstrap resampling procedure for model building: application to the Cox regression model. Stat Med 1992 11 2093 109 1293671 10.1002/sim.4780111607

[B10] Royston P Sauerbrei W Stability of multivariable fractional polynomial models with selection of variables and transformations: a bootstrap investigation Stat Med 2003 22 639 59 10.1002/sim.1310 12590419

[B11] Augustin NH Sauerbrei W Schumacher M The practical utility of incorporating model selection uncertainty into prognostic models for survival data Statistical Modelling 2005 5 95 118 10.1191/1471082X05st089oa

[B12] Donders AR van der Heijden GJ Stijnen T Moons KG Review: a gentle introduction to imputation of missing values J Clin Epidemiol 2006 59 1087 91 10.1016/j.jclinepi.2006.01.014 16980149

[B13] Moons KG Donders RA Stijnen T Harrell FE Jr Using the outcome for imputation of missing predictor values was preferred J Clin Epidemiol 2006 59 1092 101 10.1016/j.jclinepi.2006.01.009 16980150

[B14] Heymans MW van Buuren S Knol DL van Mechelen W de Vet HC Variable selection under multiple imputation using the bootstrap in a prognostic study BMC Med Res Methodol 2007 7 33 10.1186/1471-2288-7-33 17629912 PMC1945032

[B15] Kuijpers T van der Heijden GJMG Vergouwe Y Twist JWR Boeke AJP Bouter LM van der Windt DAWM Good generalizability of a prediction rule for prediction of persistent shoulder pain in the short term J Clin Epidemiol 2007 60 947 53 10.1016/j.jclinepi.2006.11.015 17689811

[B16] Van der Heijden GJ Leffers P Bouter LM Shoulder disability questionnaire design and responsiveness of a functional status measure J Clin Epidemiol 2000 53 29 38 10.1016/S0895-4356(99)00078-5 10693901

[B17] Van der Windt DA Koes BW Devillé W Boeke AJ de Jong BA Bouter LM Effectiveness of corticosteroid injections versus physiotherapy for treatment of painful stiff shoulder in primary care: randomised trial BMJ 1998 317 1292 6 9804720 10.1136/bmj.317.7168.1292 PMC28713

[B18] Van der Roer N Ostelo RW Bekkering GE van Tulder MW de Vet HC Minimal clinically important change for pain intensity, functional status, and general health status in patients with nonspecific low back pain Spine 2006 31 578 582 10.1097/01.brs.0000201293.57439.47 16508555

[B19] Kuijpers T van der Windt DA van der Heijden GJ Bouter LM Systematic review of prognostic cohort studies on shoulder disorders Pain 2004 109 420 31 10.1016/j.pain.2004.02.017 15157703

[B20] Berg SGM Vlaeyen JWS Ter Kuil MM Spinhoven P van Breukelen G Kole-Snijders AMJ Instruments for measuring chronic pain, part 2. Pain Coping and Cognition List Dutch: Meetinstrument chronische pijn, deel 2. Pijn Coping Cognitie Lijst 2001 Maastricht: Pijn Kennis Centrum

[B21] Terluin B van Rhenen W Schaufeli W de Haan M The four-Dimensional symptom questionnaire (4DSQ): measuring distress and other mental health problems in a working population Work & Stress 2004 18 187 207

[B22] Waddell G Newton M Henderson I Somerville D Main CJ A Fear-Avoidance Beliefs Questionnaire (FABQ) and the role of fear-avoidance beliefs in chronic low back pain and disability Pain 1993 52 157 68 10.1016/0304-3959(93)90127-B 8455963

[B23] Kori SH Miller RP Todd DD Kinesiophobia: A new view of chronic pain behaviour Pain Management 1990 35 43

[B24] Vlaeyen JW Seelen HA Peters M de Jong P Aretz E Beisiegel E Weber WEJ Fear of movement/(re)injury and muscular reactivity in chronic low back pain patients: an experimental investigation Pain 1999 82 297 304 10.1016/S0304-3959(99)00054-8 10488681

[B25] Royston P Altman DG Sauerbrei W Dichotomizing continuous predictors in multiple regression: a bad idea Stat Med 2006 25 127 41 10.1002/sim.2331 16217841

[B26] Slinker BK Glantz SA Multiple regression for physiological data analysis: the problem of multicollinearity American Journal of Physiology 1985 249 R1 R12 4014489 10.1152/ajpregu.1985.249.1.R1

[B27] Akaike H Petrov BN, Csaki Fsor 2nd Int. Symp. on Information Theory 1973 Budapest: Akademiai Kiado 267 81 Bozdogan H 1987 Psychometrika 52 345-70

[B28] Peduzzi P Concato J Kemper E Holford TR Feinstein AR A simulation study of the number of events per variable in logistic regression analysis J Clin Epidemiol 1996 49 1373 9 10.1016/S0895-4356(96)00236-3 8970487

[B29] Harrell FE Jr Checklist for Authors: Statistical Problems to Document and to Avoid 2007 Vanderbilt University Department of biostatistics http://biostat.mc.vanderbilt.edu/twiki/bin/view/Main/ManuscriptChecklist

[B30] Van Buuren S Oudshoorn CGM Flexible multivariate imputation by MICE. Leiden 1999 TNO Prevention and Health http://www.multiple-imputation.com/Accessed 2007 August 14

[B31] Meng X Rubin DB Performing likelihood ratio tests with multiply-imputed data sets Biometrika 1992 79 103 111 10.1093/biomet/79.1.103

[B32] Harrell FE Jr Lee KL Mark DB Multivariable prognostic models: issues in developing models, evaluating assumptions and adequacy, and measuring and reducing errors Stat Med 1996 15 361 87 10.1002/(SICI)1097-0258(19960229)15:4<361::AID-SIM168>3.0.CO;2-4 8668867

[B33] Steyerberg EW Harrell FE Jr Borsboom GJ Eijkemans MJ Vergouwe Y Habbema JD Internal validation of predictive models: efficiency of some procedures for logistic regression analysis J Clin Epidemiol 2001 54 774 81 10.1016/S0895-4356(01)00341-9 11470385

[B34] Harell FE Jr Califf RM Pryor DB Lee KL Rosati RA Evaluating the yield of medical tests' Journal of the American Medical Association 1982 247 2543 6 10.1001/jama.247.18.2543 7069920

[B35] Austin PC Tu JV Bootstrap methods for developing predictive models The American Statistician 2004 58 131 7 10.1198/0003130043277

[B36] Beyene J Atenafu EG Hamid JS To T Sung LL Determining relative importance of variables in developing and validating predictive models BMC Med Res Methodol 2009 9 64 10.1186/1471-2288-9-64 19751506 PMC2761416

[B37] Austin PC Bootstrap model selection had similar performance for selecting authentic and noise variables compared to backward variable elimination: a simulation study J Clin Epidemiol 2008 61 1009 17 10.1016/j.jclinepi.2007.11.014 18539429

[B38] Crawford SL Tennstedt SL McKinlay JB A comparison of analytic methods for non-random missingness of outcome data J Clin Epidemiol 1995 48 209 19 10.1016/0895-4356(94)00124-9 7869067

[B39] Clark TG Altman DG Developing a prognostic model in the presence of missing data: an ovarian cancer case study J Clin Epidemiol 2003 56 28 37 10.1016/S0895-4356(02)00539-5 12589867

[B40] Van der Heijden GJ Donders AR Stijnen T Moons KG Imputation of missing values is superior to complete case analysis and the missing-indicator method in multivariable diagnostic research: a clinical example J Clin Epidemiol 2006 59 1102 9 10.1016/j.jclinepi.2006.01.015 16980151

[B41] Ambler G Omar RZ Royston P A comparison of imputation techniques for handling missing predictor values in a risk model with a binary outcome Stat Methods Med Res 2007 16 277 98 10.1177/0962280206074466 17621472

[B42] Wood AM White IR Royston P How should variable selection be performed with multiply imputed data? Stat Med 2008 27 3227 46 10.1002/sim.3177 18203127

